# Development and Characterization of Laminated Composites from Açaí Residues and Castor Oil-Based Polyurethane Matrix

**DOI:** 10.3390/polym17233219

**Published:** 2025-12-03

**Authors:** Jorge Bastos Gaby Filho, Maurício Maia Ribeiro, Douglas Santos Silva, Raí Felipe Pereira Junio, José de Ribamar Mouta Araújo, Roberto Paulo Barbosa Ramos, Sergio Neves Monteiro, Jean da Silva Rodrigues

**Affiliations:** 1 Materials Engineering Program, Federal Institute of Education, Science and Technology of Pará—IFPA, Avenida Almirante Barroso, 1155, Marco, Belém 66093-020, PA, Brazil; jorgegaby10@gmail.com (J.B.G.F.); ribamar.mouta@ifpa.edu.br (J.d.R.M.A.); roberto.ramos@ifpa.edu.br (R.P.B.R.); jean.rodrigues@ifpa.edu.br (J.d.S.R.); 2 Federal Institute of Education, Science and Technology of Pará—IFPA, Estrada do Icuí Guajará, Ananindeua 67125-000, PA, Brazil; mauricio.maia@ifpa.edu.br; 3 Department of Materials Science, Military Institute of Engineering—IME, Praça General Tibúrcio, 80, Praia Vermelha, Urca, Rio de Janeiro 22290-270, RJ, Brazil; raivsjfelipe@ime.eb.br (R.F.P.J.); sergio.neves@ime.eb.br (S.N.M.)

**Keywords:** lignocellulosic composites, vegetable polyurethane, açaí residues, sustainability, laminated panels

## Abstract

This work presents the development and characterization of laminated composite panels produced from açaí residues and fibers, incorporated into a castor oil-based vegetable polyurethane matrix. The study aimed to evaluate the potential of these Amazonian agro-industrial residues as lignocellulosic reinforcement in sustainable materials. The manufacturing process was carried out by manual lamination and cold pressing, following the recommendations of ABNT NBR 14810-2:2018. The physical (moisture, density, and swelling) and mechanical (perpendicular tensile and static flexural) properties of the resulting panels were analyzed. The results revealed an average moisture content of 6.23% and a 24 h swelling of 2.76%, which are values within and well below the regulatory limits, respectively. The perpendicular tensile strength (0.49 N/mm^2^) exceeded the minimum required value, indicating good interfacial adhesion and internal cohesion. However, the flexural strength and modulus of elasticity (2.4 N/mm^2^ and 1323 N/mm^2^) were below the standards due to the absence of oriented fibers and density heterogeneity. It is concluded that the composite has high potential for indoor applications with low structural stress, standing out for its lightness, dimensional stability and environmental viability in the use of açaí residues.

## 1. Introduction

In recent decades, advances in the development of sustainable composite materials have been driven by the need to reduce dependence on petroleum-based synthetics and to mitigate environmental impacts associated with the improper disposal of agro-industrial residues [1,2]. Within this scenario, the valorization of lignocellulosic by-products from tropical crops has emerged as a promising route for producing low-cost, renewable, and eco-efficient composites with good mechanical and physical performance [3,4]. Among these residues, the açaí (*Euterpe oleracea* Mart.) seed stands out as one of the most abundant agro-industrial wastes in the Amazon region, representing over 80% of the total fruit mass and generating thousands of tons of discarded material annually, often without proper management [5,6].

The lignocellulosic composition of açaí residues—rich in cellulose, hemicellulose, and lignin—provides mechanical strength and chemical functionality suitable for reinforcement in polymer matrices [7,8]. Previous research on natural fillers such as babassu, coconut, rice husk, and Brazil nut shells demonstrated the feasibility of using agricultural by-products as reinforcement for polymer composites [9,10]. However, studies involving açaí fibers and particles are still scarce, especially in laminated or particleboard configurations, which represents an existing technological and scientific gap [11,12].

In this context, the combination of açaí residues with bio-based polymeric matrices represents an innovative and sustainable route for the development of new materials aligned with the principles of the circular bioeconomy [13]. Particularly, polyurethanes derived from vegetable oils, such as castor oil (*Ricinus communis*), have gained attention due to their renewable origin, low toxicity, and excellent adhesion to lignocellulosic substrates [14,15,16,17]. Several studies have reported the successful use of castor oil-based polyurethane in the production of natural fiber composites, demonstrating improved interfacial adhesion, dimensional stability, and mechanical strength under controlled processing conditions [18,19,20].

Beyond its environmental motivation, the present work also advances the state of the art by employing açaí seed residues in a laminated composite architecture, which remains scarcely explored in the literature. Although açaí biomass has been investigated in other polymeric matrices—such as its use as reinforcement in thermoplastic and biodegradable systems [5], in PBAT biocomposites [8], and in lignocellulosic agro-waste composites [9,10,11,12]—these studies typically focus on homogeneous particleboards, extrusion-processed composites, or molded particulate panels. In contrast, the present research incorporates both açaí fibers and seed particles into a resin-impregnated laminated structure using a castor oil-based polyurethane matrix, representing an innovative configuration not previously reported. This laminated architecture broadens the technological possibilities for açaí-based materials by enabling improved interfacial contact, better matrix encapsulation of residues, and new design routes for sustainable engineered panels.

The sustainability and versatility of vegetable-based polyurethanes also extend to their non-isocyanate synthesis routes, reducing the use of toxic reagents and promoting safer processing [21]. Furthermore, bio-based polyols synthesized from castor, rapeseed, or soybean oils have shown potential to replace fossil-derived polyols in rigid and flexible polyurethane systems [22,23]. These matrices exhibit good thermal and mechanical performance, as well as resistance to moisture absorption, which are desirable properties for indoor and low-load structural applications [24].

The Amazonian context of this research adds an important socio-environmental dimension. The reuse of açaí residues, one of the main organic liabilities of the region, contributes to reducing environmental pollution while generating new technological and economic opportunities for local communities [25]. Such initiatives reinforce the role of bio-based materials in supporting the United Nations Sustainable Development Goals (SDGs), particularly those related to responsible consumption, innovation, and climate action [26].

Therefore, this study proposes the development and characterization of laminated composites made from açaí seed residues and fibers incorporated into a castor oil-based polyurethane matrix. The manufacturing process was designed to be environmentally friendly, using manual lamination and cold pressing, avoiding solvents and minimizing energy consumption. The physical (moisture, density, and swelling) and mechanical (perpendicular tensile and flexural) properties of the resulting panels were evaluated to verify their technical feasibility, dimensional stability, and internal cohesion. The results are expected to demonstrate that the combination of Amazonian agro-residues with a bio-based polymer matrix can generate lightweight, cohesive, and sustainable composites, capable of partially replacing conventional materials in indoor applications with low structural stress.

It is important to emphasize that the present study is strictly aligned with the performance requirements established by ABNT NBR 14810-2:2018 [27], which regulates particleboard and laminated lignocellulosic panels intended for indoor, low-temperature, non-structural applications. Consequently, the properties that govern real in-service performance are moisture behavior, dimensional stability, density uniformity, internal bonding and flexural response. Advanced thermal, structural or chemical techniques—including TGA/DTG, DSC, FTIR or XRD—while scientifically valuable, are not required for this category of materials and would not modify the engineering interpretation or the serviceability limits relevant to the target application. For this reason, the scope of the present work is intentionally focused on the normative physical and mechanical properties that determine the functional feasibility of açaí-based laminated panels in indoor environments.

## 2. Materials and Methods

### 2.1. Materials

**Figure 1:**
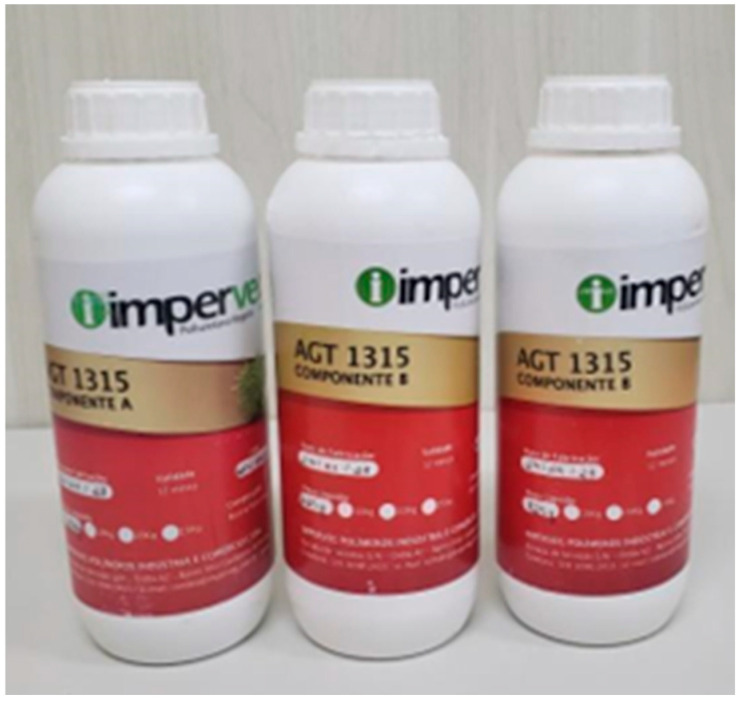
Two-component resin based on vegetable polyurethane derived from castor beans.

In the fabrication of the laminated composite panels, açaí seeds and a castor oil-based bicomponent resin were employed as the main constituents. The açaí seeds, derived from agro-industrial waste generated after the pulp extraction process, were collected from a local vendor located in the Umarizal district of Belém, Pará, Brazil. The Imperveg AGT 1315 resin (Figure 1), supplied by Imperveg Polímeros Indústria e Comércio Ltd. (Aguaí, São Paulo, Brazil), is a vegetable polyurethane two-component system synthesized from castor oil and is entirely free of solvents and toxic vapors. It is formulated through the cold mixing of a prepolymer with a polyol, resulting in a material with high physicochemical stability, good elasticity, and excellent impermeability. These characteristics make it particularly suitable for use as a polymeric matrix in structural composites, providing enhanced mechanical strength, dimensional integrity, and environmentally sustainable performance.

### 2.2. Methods

#### 2.2.1. Collection and Drying of Açaí Seeds

**Figure 2:**
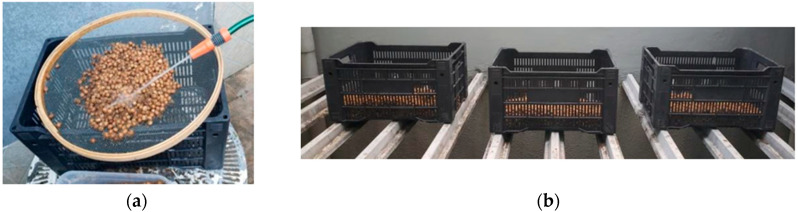
Collection and drying: (**a**) Hand washing; (**b**) Natural drying.

After collection, the açaí seeds were first weighed using a precision digital scale (model OMRON HN-269) to record their initial mass. Subsequently, they were manually washed under running water using a metal sieve with mesh no. 55 to remove organic residues and surface impurities. After cleaning, the seeds were placed in perforated plastic trays to allow proper air circulation and were air-dried under ambient conditions for 36 h, as illustrated in Figure 2.

**Figure 3:**
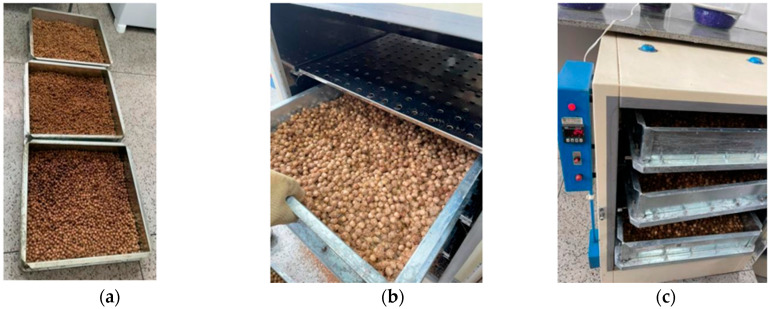
Oven drying: (**a**) Storage; (**b**) and (**c**) Insertion in oven.

Subsequently, the seeds were transferred to the Food Inspection and Bromatology Laboratory (LIAB/8) of the 8th Supply Battalion (8º BSup), Brazilian Army, for the oven-drying stage. The process was carried out in a forced-air circulation oven (model Lucadema 64 L) at 50 °C for 72 h, with manual agitation of the particles twice a day to ensure uniform drying (Figure 3). From the initial 20.036 kg of wet seeds collected, a total of 11.63 kg of oven-dried material was obtained, corresponding to a mass reduction of 41.95%, attributed to the moisture loss from the seeds.

#### 2.2.2. Fiber Extraction

**Figure 4:**
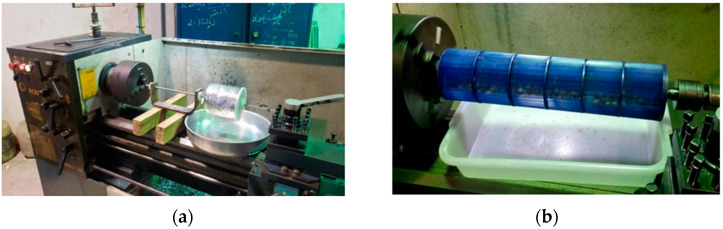
Mechanical extraction of fibers: (**a**) Prototype 01; (**b**) Prototype 02.

Two fiber extraction methods were evaluated: mechanical and manual. For the mechanical method, two defibrillation prototypes were developed, each consisting of a perforated metallic chamber coupled to a mechanical rotor, designed to promote friction and facilitate the separation of fibers from the açaí seeds (Figure 4). Prototype 01 yielded an average of 0.47 g of fiber per 100 g of seeds when operated at 80 rpm for 40 min, whereas Prototype 02 produced a higher yield of 0.88 g/100 g under the same operating conditions, demonstrating greater defibrillation efficiency compared to the first design.

**Figure 5:**
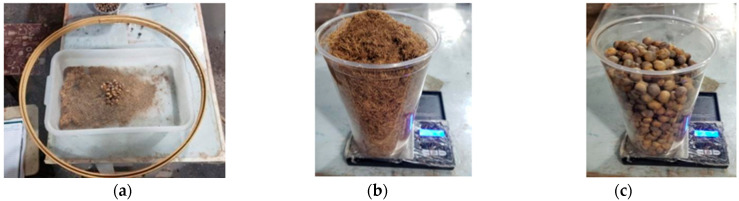
Fiber separation: (**a**) Manual extraction; (**b**) Extracted fibers; (**c**) Residual seeds.

Due to the low yield obtained with the mechanical method, the manual fiber extraction procedure was adopted, using a metal sieve with mesh no. 55 (Figure 5). The process was carried out over a seven-day period, with three hours of manual operation per day, yielding 62.21 g of fibers from 1000 g of seeds, leaving 912.95 g of residual kernels after processing. For the manufacture of the final composite panels, a total of 3266.60 g of oven-dried açaí seeds was used, consisting of 198.13 g of extracted fibers and 3006.05 g of residual kernels incorporated into the process.

#### 2.2.3. Seed Grinding

**Figure 6:**
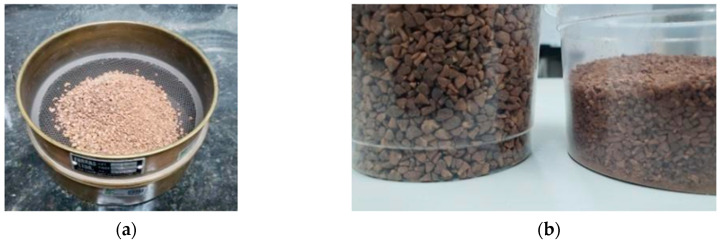
(**a**) Sieves used; (**b**) Differences in the particle size of the residues.

The collected seeds were ground using a WILEY 500 knife mill (LOGEN) to achieve particle size reduction and homogenization. The milled material was subsequently sieved through a metallic screen USS No. 10—Tyler 9 (2.0 mm opening), producing three fractions with distinct particle size distributions (Table 1). Only the fractions with suitable granulometry were selected for the manufacture of the composite panels (Figure 6).

#### 2.2.4. Characterization of Fibers and Seeds

The physical and morphological characterization of fibers and untreated açaí seeds was carried out at the Scanning Electron Microscopy (SEM) Laboratory of the Federal Institute of Pará (IFPA). The analyses were performed using a VEGA 3 LMU scanning electron microscope equipped with an energy-dispersive X-ray spectroscopy (EDS) system. Samples were gold-sputtered for 210 s under a current of 20 mA to ensure adequate surface conductivity for observation. This procedure enabled a detailed assessment of the morphology, homogeneity, and structural integrity of the fibers and seeds, as well as the identification of potential elemental contaminants within the samples.

#### 2.2.5. Manufacturing of Laminated Composite Panels

The manufacturing of the laminated composite panels was carried out in accordance with the Brazilian Standard ABNT NBR 14810-2:2018 [27], which classifies type P2 panels as suitable for indoor use under dry conditions, with a nominal thickness between 13 and 20 mm. Initially, two experimental prototypes (PT1 and PT2) were fabricated to evaluate the processing procedures and optimize the distribution of fibers, ground seed particles, and polymeric matrix. These prototypes served as a basis for refining the mixing, compaction, and curing parameters, ensuring the reproducibility and quality of the final panels.

##### Test Panel 1 (PT1)

**Figure 7:**
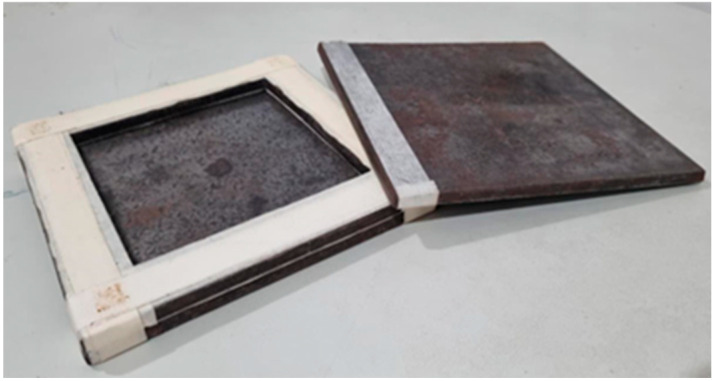
Mold used for Test Panel 1 (PT1).

**Figure 8:**
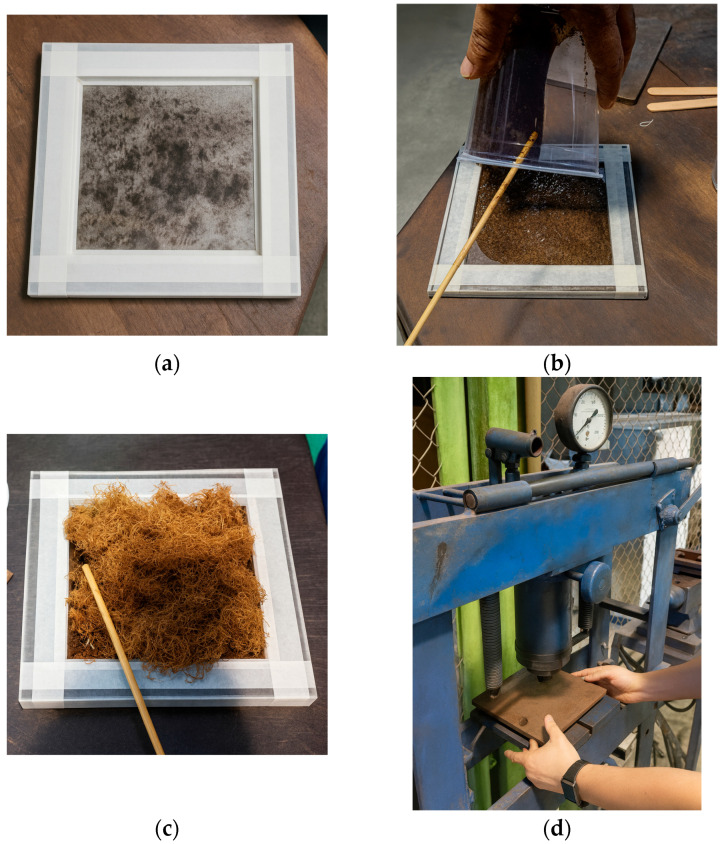
PT1: (**a**) Mold; (**b**) Placement of crushed waste and resin; (**c**) Insertion of fibers with resin; (**d**) Placement in the press.

**Figure 9:**
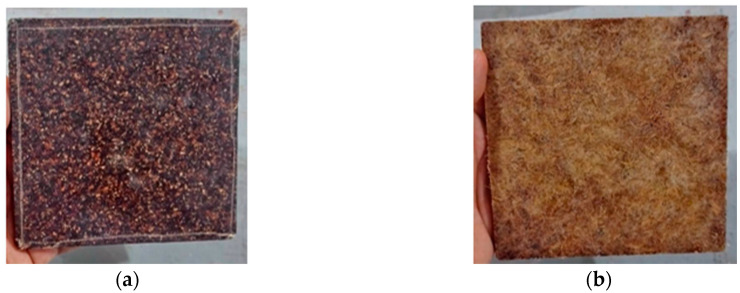
PT1—Finishing: (**a**) Main face; (**b**) Rear face.

For the fabrication of Test Panel 1 (PT1), a metal mold measuring 120 mm × 120 mm × 7 mm (Figure 7) was used, sealed with metallic plates and previously coated with a release wax to facilitate demolding after curing. Initially, ground seed residues with particle size passing through a Tyler 9 metallic sieve (2.0 mm) were applied to form the front surface of the panel, followed by the partial deposition of the polymeric resin. Subsequently, vegetable fibers were added and covered with the remaining fraction of resin. The assembly was cold-pressed under a load of 2 tons and allowed to cure until the prototype was fully consolidated (Figure 8). This procedure enabled the assessment of the molding feasibility and the preliminary evaluation of the physical and aesthetic characteristics of the resulting panel (Figure 9).

##### Test Panel 2 (PT2)

**Figure 10:**
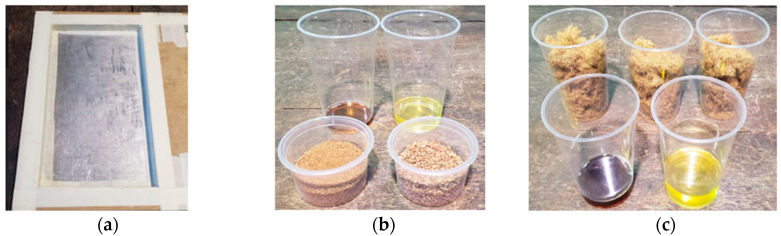
PT2: (**a**) Mold; (**b**) Resin to be mixed with crushed seeds; (**c**) Resin to be mixed with fibers.

**Figure 11:**
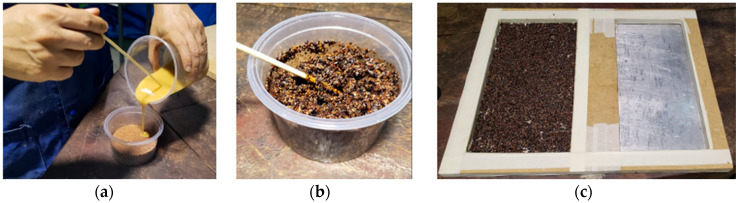
PT2: (**a**) Mixing process; (**b**) Mixed materials; (**c**) Application to the mold.

Aiming to improve the manufacturing process, Test Panel 2 (PT2) was fabricated using a plywood mold measuring 120 mm × 315 mm × 15 mm, adapted from the final model design. The mold was treated with release wax and sealed with masking tape to ensure proper edge integrity and facilitate demolding after curing. The panel was composed of 227.06 g of açaí seed particles—half retained and half passing through a Tyler 9 sieve (2.0 mm)—together with 28.12 g of fibers and 301.56 g of castor oil-based polyurethane resin (Figure 10). The lamination process was carried out in successive layers, with fine particles forming the front surface, fibers placed in the intermediate layer, and coarser particles arranged in the back layer (Figure 11), aiming to achieve better constituent distribution and enhanced interfacial bonding within the composite structure.

**Figure 12:**
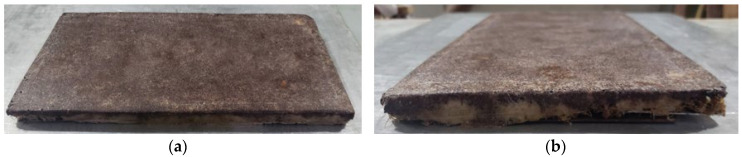
PT2: (**a**) Front face; (**b**) Detail of the front face.

**Figure 13:**
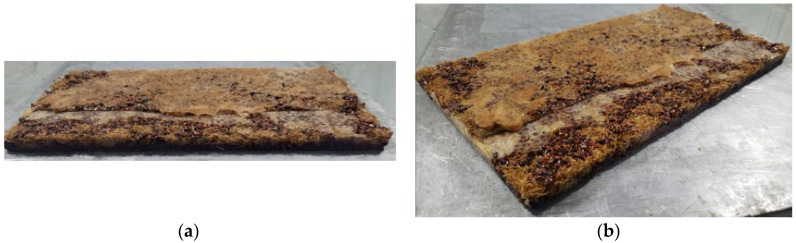
PT2: (**a**) Back face; (**b**) Detail of the back face.

The panel was cold-pressed under a load of 2 tons for 24 h. All panels were pressed under ambient laboratory conditions, with temperature maintained at 24–26 °C and relative humidity between 55–65%, which corresponds to the standard curing environment recommended for castor oil-based polyurethane systems. Pressing was performed at 2 tons of applied load using a cold-press configuration, without external heating. After pressing, each panel remained under constant load for 24 h, ensuring complete ambient-temperature curing of the polyurethane. No post-curing or thermal treatment was applied, as the resin system is formulated for full polymerization under room-temperature and moderate-humidity conditions. After demolding, the front surface exhibited a satisfactory finish, whereas the back surface showed slight deformations and irregularities, suggesting the need for adjustments in compaction parameters and resin distribution during the forming process (Figure 12 and Figure 13).

##### Final Panels (PD1 and PD2)

**Figure 14:**
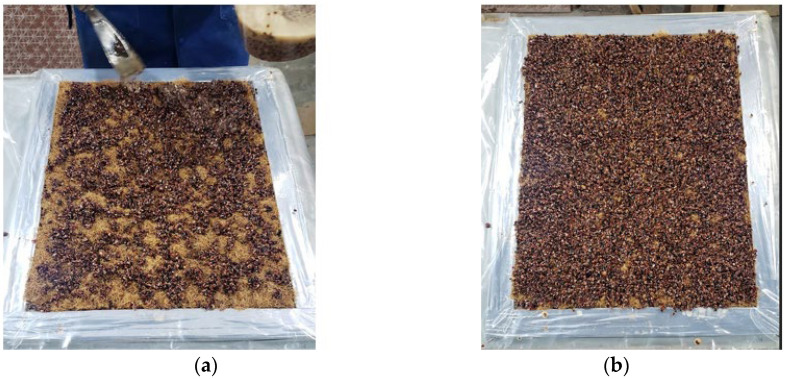
Final Panel PD1—(**a**) Manufacturing and (**b**) Placement of materials.

The final composite panels (PD1 and PD2) were fabricated using a plywood mold measuring 415 mm × 315 mm × 15 mm, dimensioned to allow the extraction of standard test specimens for subsequent characterization. The manufacturing process followed the same procedure used for Test Panel 2 (PT2), including the application of release wax, edge sealing with plywood sheets, and cold pressing under a load of 2 tons for 24 h. The formulation of the composite was previously defined, consisting of 97.77 g of fibers, 789.51 g of açaí seed particles—50% retained and 50% passing through a Tyler 9 sieve (2.0 mm)—and 1048.56 g of castor oil-based polyurethane resin, composed of 419.42 g of component A and 629.14 g of component B. The lamination process was carefully performed to ensure composite homogeneity and uniform distribution of all constituents (Figure 14).

It is also important to clarify that the resin content in the final panels corresponds exactly to the mass introduced during lamination, since the cold-cure polyurethane system used does not bleed, volatilize, or undergo mass loss during consolidation. Because the mold is fully sealed and no overflow occurs under the applied pressing conditions, the final composite retains 100% of the resin originally weighed. For panels PD1 and PD2, the final formulation therefore contained approximately 52 wt% polyurethane resin and 48 wt% açaí residues (fibers + particles). This proportion governs the composite’s mechanical response and is consistent with the requirements of ABNT NBR 14810-2:2018 [27] for indoor, particleboard-type panels.

**Figure 15:**
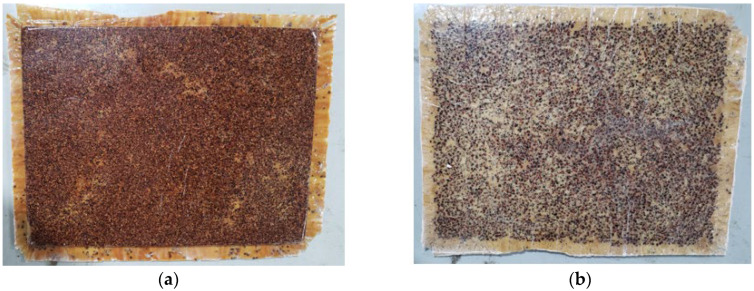
Final Panel PD1 with trimmed burrs: (**a**) Front face; (**b**) Back face.

After demolding, the final panels PD1 and PD2 were edge-trimmed using a micro rotary grinder until reaching the dimensions specified in the cutting plan. Both panels exhibited well-defined front and back surfaces with good surface uniformity, as shown in Figure 15.

#### 2.2.6. Specimen Fabrication and Characterization

**Figure 16:**
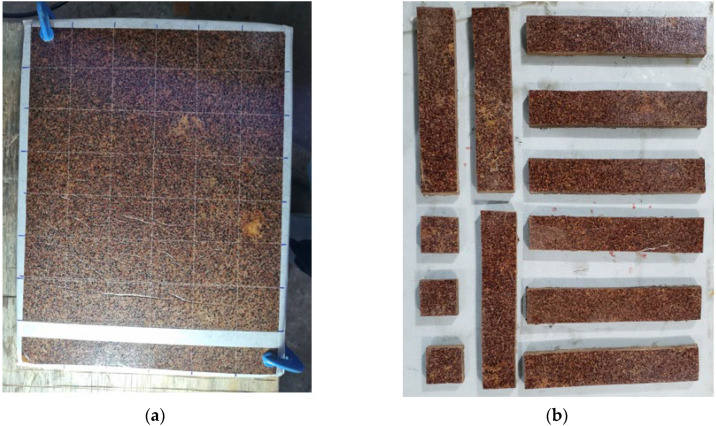
Test specimens: (**a**) Pre-marked PD1; (**b**) Extracted test specimens PD2.

After the pressing and curing stages, the composite panels were sectioned into test specimens in accordance with the dimensions and specifications defined by the Brazilian Standard ABNT NBR 14810-2:2018 [27], applicable to particleboard panels used for mechanical and physical testing. This initial preparation step is illustrated in Figure 16, which shows the cutting process performed to obtain the experimental samples used in the subsequent analyses.

##### Moisture Content Determination

The moisture content test was conducted at the Chemistry Laboratory of the Federal Institute of Pará (IFPA), Belém Campus. A total of ten specimens with dimensions of 50 mm × 50 mm × 15 mm, obtained from the final panel (PD1), were used in the analysis. Initially, the wet mass of the specimens was recorded, after which they were oven-dried at 103 °C until constant mass was achieved, following standard procedures for lignocellulosic panels. After cooling in a desiccator, the dry mass was measured using a Shimadzu precision digital balance, allowing the calculation of the moisture variation in the samples.

##### Density Determination

The apparent density test was performed at the Chemistry Laboratory of the Federal Institute of Pará (IFPA), Belém Campus. A total of ten specimens with dimensions of 50 mm × 50 mm × 15 mm, obtained from the final panel (PD1), were evaluated. The mass of each specimen was measured using a Shimadzu precision digital balance, and the dimensions (central thickness, width, and length) were determined with a digital caliper, allowing the calculation of the individual density of each sample. For comparison purposes, the density range of type P2 MDF (650 to 750 kg/m^3^) was adopted as a reference, representing materials intended for indoor applications under dry conditions and dimensionally comparable to the composite panels produced in this study.

##### Perpendicular Tensile Strength Determination

**Figure 17:**
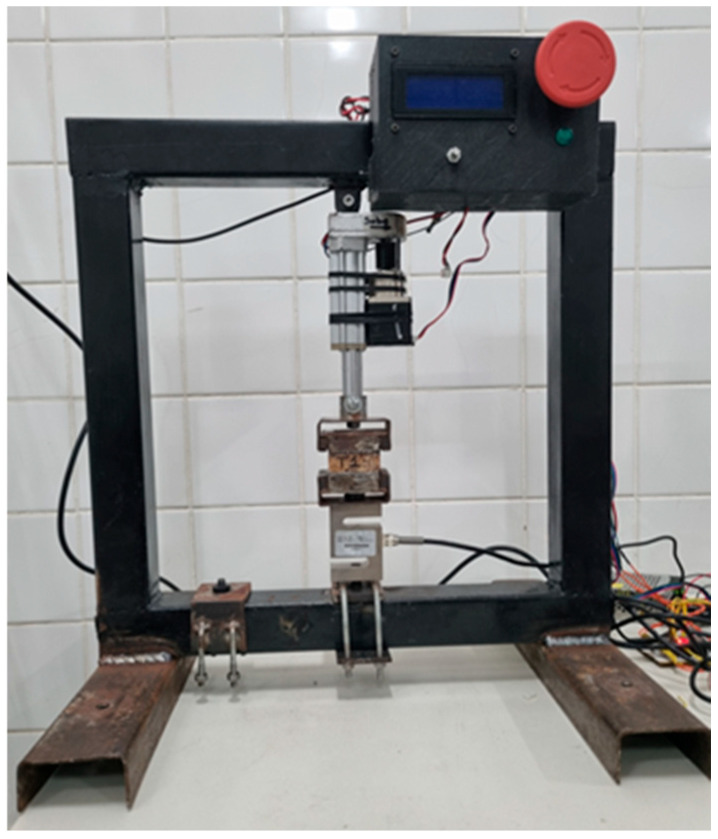
Equipment developed for performing perpendicular tensile tests.

The perpendicular tensile strength test was carried out using ten specimens with dimensions of 50 mm × 50 mm × 15 mm, obtained from the final panel (PD1). For this purpose, a custom testing apparatus was designed and built at the Materials Characterization Laboratory of the Federal Institute of Pará (IFPA), Belém Campus (Figure 17). The experimental setup consisted of a carbon steel frame, a load cell with a capacity of 1 tf (≈9.81 kN), a linear actuator with a 50 mm stroke, a signal amplifier (ADS-1232), and a stepper motor, all integrated into a dedicated control system developed for this test. This configuration allowed controlled load application and accurate recording of force and displacement data throughout the experiment.

**Figure 18:**
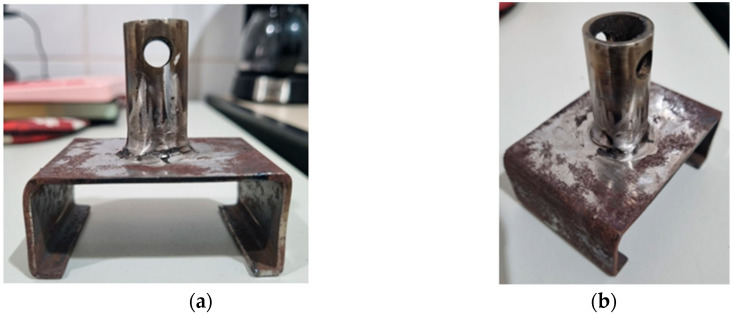
Specimen clamping device: (**a**) Front view; (**b**) Perspective.

**Figure 19:**
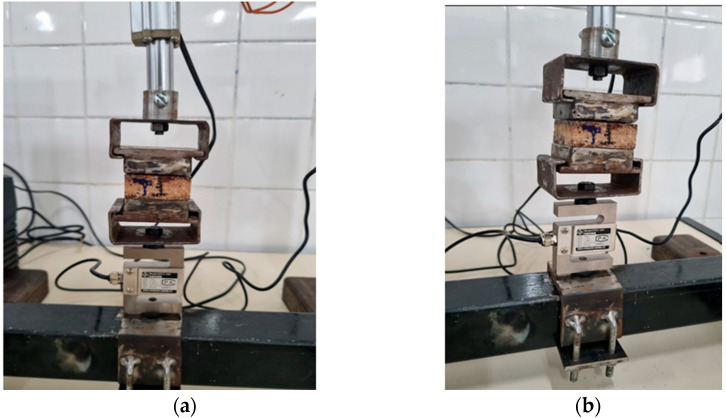
Assembly attached to the equipment: (**a**) Front view; (**b**) Perspective.

For specimen fixation during testing, two metallic fixtures were fabricated (Figure 18) in accordance with the dimensional specifications established by the reference standard. In addition, twenty auxiliary metallic plates were produced to enable bonding of the specimens using a medium-viscosity instant adhesive (Teckbond 793). After applying the adhesive, the assemblies were subjected to a compressive load of 20 kg for 24 h to ensure complete curing and uniform adhesion between the bonded surfaces. Subsequently, the prepared fixtures were mounted onto the testing machine, allowing controlled application of perpendicular load and accurate recording of the tensile strength values (Figure 19).

##### Static Bending Strength and Modulus of Elasticity Determination

**Figure 20:**
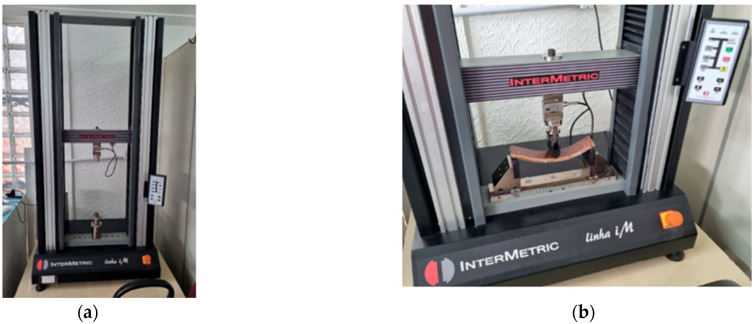
Universal Testing Machine: (**a**) General view; (**b**) Detail.

The static bending test, aimed at determining the flexural strength and modulus of elasticity (MOE), was performed using ten specimens with dimensions of 220 mm × 50 mm × 15 mm, obtained from the final panel (PD2). The experiments were conducted at the Laboratory of the Federal University of Pará (UFPA), located in Ananindeua, Pará, Brazil, using an iM50 Electromechanical Universal Testing Machine with a 50 kN capacity, equipped with a 5 kN load cell and operated at a constant crosshead speed of 5 mm/min (Figure 20).

##### Thickness Swelling Determination After 24 h

The 24 h thickness swelling test was performed to evaluate the dimensional stability of the composite panels under water immersion conditions. A total of ten specimens measuring 50 mm × 50 mm × 15 mm, obtained from the final panel (PD1), were used in the analysis. The central thickness of each specimen was measured using a precision digital caliper, before and after 24 h of water immersion at room temperature. The dimensional variation was expressed as the percentage of thickness swelling, calculated from the difference between the initial and final thickness values.

## 3. Results and Discussion

Before presenting the experimental results, it should be clarified that the present composite is designed for indoor applications under ambient temperature and humidity conditions, as defined by ABNT NBR 14810-2:2018 [27]. For this class of materials, performance is determined by moisture content, dimensional stability, density uniformity, internal bonding, and flexural behavior, rather than by thermal degradation phenomena. For this reason, advanced techniques such as TGA/DTG, DSC, FTIR and XRD were not included. The polyurethane used is a commercial bicomponent system with predefined stoichiometry and complete curing at room temperature, making analyses of Tg, residual cure, crosslink density, hydrogen bonding, or crystallinity unnecessary for evaluating serviceability. Moreover, the açaí residues were processed into short fibers and particles, eliminating long-range cellulose order and limiting the relevance of crystallinity measurements. Thus, the experimental program focuses exclusively on the properties required by the standard and directly linked to the panel’s intended application, without compromising the scientific or engineering validity of the results.

### 3.1. Characterization of Açaí Fibers and Seeds

#### 3.1.1. Morphology

**Figure 21:**
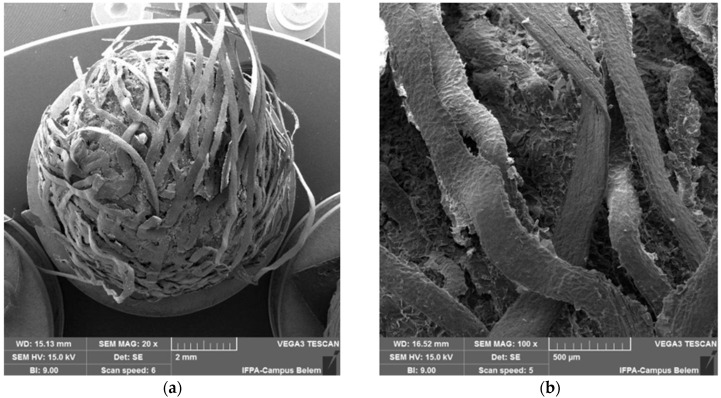
Scanning electron microscopy (SEM) micrographs of fresh açaí seed: (**a**) general view of the fibrous structure of the seed (20×); (**b**) surface detail showing fiber bundles with rough texture and the presence of cavities and irregularities typical of lignocellulosic materials (100×).

Figure 21 shows scanning electron microscopy (SEM) micrographs of the raw açaí seed, highlighting its characteristic fibrous structure.

At 20× magnification, the açaí seed appears as a roughly spherical structure covered by intertwined, ribbon-like lamellae arranged in a helical or coiled pattern. These overlapping strips form a dense endocarpic shell, evidencing anisotropy—the fiber orientation and fracture paths suggest that mechanical resistance is higher tangentially to the lamellae and weaker radially. The overall surface is rough and scaly, with clear delamination zones, lifted edges, and broken flakes, indicative of moderate toughness and fracture mechanisms involving pull-out and interlaminar debonding. Given the 2 mm scale bar, the visible seed portion spans several millimeters in diameter, consistent with the hard endocarp typical of tropical drupes. The lamellae themselves have submillimetric thickness, suggesting a multilayered structure with a gradient of porosity from the exterior surface inward.

At 100× magnification, these lamellae are resolved as flattened, twisted fiber bundles. The surfaces exhibit intrinsic roughness, characterized by longitudinal striations, grooves, and scaly microtextures—typical of lignocellulosic matrices, where cellulose microfibrils are embedded in a heterogeneous amorphous matrix of hemicellulose and lignin. The width of individual bundles is approximately 100–300 µm, and the interbundle regions are filled with fine particulate residues, possibly remnants of the mesocarp or degraded lignocellulosic fragments. Numerous microcracks traverse the lamellae, often aligned with the fibrillar direction, indicating preferred crack propagation paths through cleavage and interfacial decohesion.

#### 3.1.2. Chemical Composition

**Figure 22:**
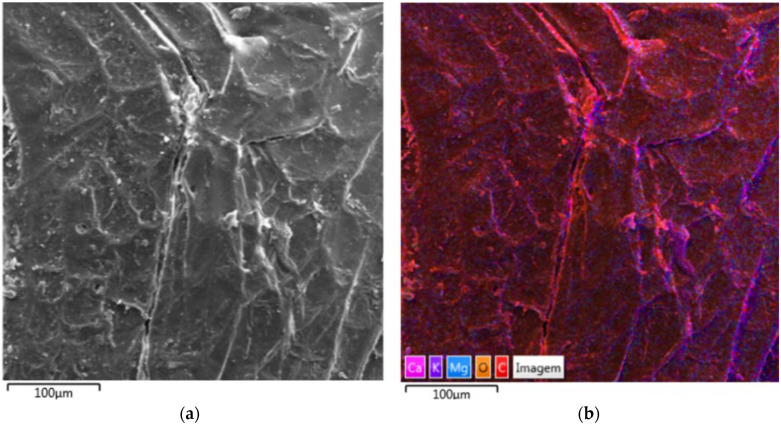
Scanning electron microscopy (SEM) micrographs of fresh açaí seeds: (**a**) secondary electron image, showing the surface morphology and the presence of fissures and compacted regions; (**b**) chemical mapping by energy dispersive spectroscopy (EDS), showing the elemental distribution of Ca, Mg, O and C on the surface, associated with the lignocellulosic composition and the presence of natural mineral traces in the material.

The micrographs presented in Figure 22 correspond to the surface of the natural açaí seed, observed by scanning electron microscopy (SEM) with secondary electrons (Figure 22a) and mapping by energy dispersive X-ray spectroscopy (EDS) in elemental overlay mode (Figure 22b).

Morphological analysis reveals a typical lignocellulosic surface, with irregular topography, the presence of intertwined ridges and microtracks, and multiple fine cracks and delaminations. These discontinuities indicate a natural composite structure, in which cellulose microfibrils are embedded in an amorphous matrix composed of hemicellulose and lignin. The preferred direction of the observed striations suggests intrinsic anisotropy, with greater strength along the fibers and fragility at the interfaces between the lamellae. Small particles or agglomerates adhered to the surface may correspond to mineral residues or natural ash trapped in the microcracks.

EDS mapping reinforces this interpretation by highlighting the spatial distribution of the main constituent elements. Carbon (C) and oxygen (O) are widely distributed throughout the surface, confirming the organic and lignocellulosic nature of the matrix. In contrast, the elements calcium (Ca), potassium (K), and magnesium (Mg) appear concentrated in specific regions, forming small areas of enrichment coinciding with the cracks and junctions between the lamellae. This localized concentration suggests the presence of mineral salts, such as oxalates, carbonates, or phosphates, naturally incorporated into the plant tissue. It is also possible that some of these elements are associated with ion exchange processes, with cations adsorbed on functional groups of lignins or pectins, in addition to possible surface deposition of minerals from the soil or water during fruit growth.

It is important to emphasize that EDS mapping provides a qualitative and semi-quantitative analysis of the composition, requiring caution in interpretation. The carbon signal may be overestimated due to carbon metallization or the use of carbon-based conductive tapes. Furthermore, the electron interaction volume at 15 kV spans a few micrometers in depth, mixing information from the surface and subsurface layers. Accurate quantification requires matrix effect corrections (MAFs) and the use of appropriate standards.

In general, the images indicate that the açaí seed has a heterogeneous, fibrous, and slightly porous surface, composed primarily of carbon and oxygen, with small mineral inclusions of calcium, potassium, and magnesium concentrated in zones of structural discontinuity. This configuration reveals an anisotropic natural material, whose microstructure directly influences its mechanical, thermal, and chemical behavior.

**Figure 23:**
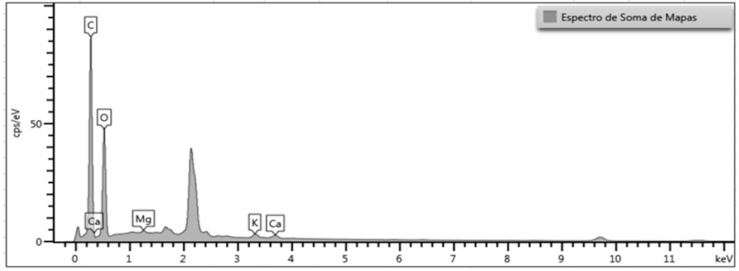
Spectrum obtained by energy dispersive spectroscopy (EDS) of raw açaí fiber, highlighting the main chemical elements present in the sample.

Figure 23 shows the EDS spectrum obtained for raw açaí fiber, in which it is possible to identify the major elements carbon and oxygen, characteristic of the lignocellulosic constitution of the material.

The EDS (sum of maps) spectrum of the in natura açaí fiber reveals an essentially organic substrate with C (≈0.28 keV) and O (≈0.53 keV) as dominant peaks, consistent with a lignocellulosic matrix (cellulose/hemicellulose/lignin). The high C/O ratio indicates polymeric fractionation typical of plant tissues and, from a thermal point of view, anticipates pyrolysis behavior with a high volatile fraction. Discrete peaks of Ca (line L ~0.34 keV and Kα ~3.69 keV), K (Kα ~3.31 keV), and Mg (Kα ~1.25 keV) appear on this organic continuum, confirming intrinsic and/or adsorbed mineral ash. These alkaline/alkaline earths tend to be located in lamellae, cracks, and middle lamella, where pectins and lignin chelate cations, and are associated with osmotic regulation and parietal tissue reinforcement.

The pronounced peak near ~2.1 keV and the small hump around ~9.7–10 keV suggest gold (M and L lines) from metallization, not belonging to the material. This is important because: (i) it confirms adequate conductive preparation; (ii) it explains part of the continuum in the 1.8–2.4 keV sector; (iii) it is a reminder that these signals should not be included in the quantification of native minerals. Similarly, C may be overestimated if carbon ribbon was present; in this case, quantification should be done with ZAF/Φ(ρz) corrections and/or background subtracted by ribbon blank.

Among the detected minerals, K and Ca appear with relatively clean K peaks, but there is spectral interference: Kβ (3.59 keV) approaches Ca Kα (3.69 keV), requiring adequate deconvolution to avoid overestimating Ca. Mg is weaker, but consistent with trace levels of hemicellulose/pectates. In terms of detection limits, under these conditions (≈15 kV, detector with polymeric window), levels on the order of 0.1–0.3% by weight can already emerge as discrete peaks; nevertheless, the associated maps are more reliable for spatial distribution than for absolute content.

It is important to note that the EDS results presented in this study are intended only as qualitative confirmation of the elemental composition typically associated with lignocellulosic biomass. Because EDS provides localized, semi-quantitative information dependent on the electron interaction volume, the technique cannot be used to determine bulk composition or absolute mineral content. The detected elements (C, O, Ca, K, Mg) are consistent with natural mineral traces commonly reported in plant-based residues and do not affect the mechanical behavior of the composite. For this reason, and because ABNT NBR 14810-2:2018 [27] does not require full chemical quantification for indoor particleboard-type panels, additional methods such as XRF or ICP-OES were not employed. The EDS analysis therefore serves exclusively to support the morphological observations and verify the presence of expected elemental constituents.

### 3.2. Characterization of Composites

#### 3.2.1. Moisture Content

Table 2 presents the results of the moisture content of the evaluated test specimens according to ABNT NBR 14810-2:2018 [27].

The results obtained for the ten test specimens showed moisture content values ranging from 5.85% to 8.98%, with an average value of 6.23 ± 1.16%. This variation range is quite narrow, less than 1 percentage point, demonstrating high uniformity among the samples and good control of the manufacturing process. This homogeneity indicates that the drying and curing process of the panels was efficient, ensuring uniform moisture distribution throughout the composite structure. The average result obtained is fully within the range established by the standard (5% to 13%), characterizing the material in a state of hygroscopic equilibrium suitable for indoor use. This finding reinforces that the composite has reached a stable state in terms of water content, an essential condition for the reliability of subsequent physical and mechanical tests.

From a technical standpoint, the average value of 6.2% suggests good compatibility between the açaí residue and the castor oil polyurethane. Lignocellulosic residue naturally tends to absorb water due to the presence of hydroxyl (–OH) groups in cellulose and hemicellulose. However, the polyurethane matrix, formed from castor oil, acts as a diffusion barrier that limits moisture penetration, reducing the composite’s overall hygroscopicity. This balance between the reinforcement and matrix phases is indicative of efficient interfacial adhesion, a result that may be associated with both the polyurethane formulation and the adequate dispersion and incorporation of the residue particles during the lamination process. Thus, the low moisture content observed confirms that the material does not exhibit excessive water retention, which helps minimize the risk of swelling, delamination, or loss of mechanical integrity over time.

Comparatively, moisture content values between 6% and 9% are common in lignocellulosic composites produced with castor oil polyurethane, while materials without a polymer matrix reach levels above 10% or 12%, depending on the relative humidity of the environment. Thus, the result obtained aligns with expectations for this type of hybrid system, confirming the effectiveness of the castor oil polymer as an encapsulating agent and hygroscopic protection for plant particles. The observed stability also indicates that the curing process was adequate, avoiding the formation of solvent-rich regions or residual moisture, which could compromise the uniformity of properties.

#### 3.2.2. Tolerance in Relation to Average Densit

Table 3 presents the apparent density results of the test specimens, calculated according to the procedures of ABNT NBR 14810-2:2018 [27].

The average density was 566.59 ± 32.50 kg/m^3^, corresponding to a coefficient of variation of approximately 5.8%. These initial values indicate that, statistically speaking, the set presents a relatively controlled overall dispersion, but individual analysis of the specimens reveals significant deviations from the normative limit.

According to the standard’s permitted range, the samples should have a density between 517 and 751 kg/m^3^. However, some specimens were found to have densities below 490 kg/m^3^, while others exceeded 600 kg/m^3^, resulting in a maximum modulus variation of 33%, as shown in Table 2. This value is well above the established limit and indicates non-compliance with the tolerance criteria. In practical terms, this demonstrates that, although the batch average is adequate, there is considerable local heterogeneity in the panel structure, reflecting an uneven resin distribution and compaction during molding.

The process used, manual lamination combined with mechanical compression, is effective in reducing overall porosity and increasing lignocellulosic particle impregnation, but it can generate density gradients between the surface layers and the central region. During the pressing stage, some of the resin tends to migrate to the peripheral zones, especially if there is excess matrix in the formulation or higher-than-necessary pressure is applied. Thus, the central regions may have a lower matrix fraction and, consequently, lower density, while the more impregnated edges become denser and more rigid. This variation is a typical phenomenon in compression molding of heterogeneous laminates, especially when the residue and polyurethane mixture is not perfectly uniformly distributed before mold closure.

Thickness variation also contributes to the observed density differences. Small fluctuations in the final thickness, resulting from the redistribution of the material under pressure, can amplify the error in determining the volume and, therefore, in the calculated density. The geometric methodology employed, based on measurements of length, width, and thickness, is sensitive to this type of variation. Therefore, it is recommended that thicknesses be measured at multiple points (at least 6 to 8 measurements per specimen) using a flat-tip micrometer with controlled contact force to accurately represent the true mean of the sample. Alternatively, complementary density determination using the hydrostatic method (Archimedes’ Principle) can provide more representative results and allow for the indirect calculation of void content by comparing it with the theoretical density obtained using the rule of mixtures.

The presence of regions with lower-than-average density may be associated with failures in the impregnation of the açaí residue by the polymer matrix, the formation of trapped air bubbles during manual lamination, or irregular retention of residual moisture prior to pressing. Denser areas tend to reflect resin buildup, indicating preferential flow of the matrix during compression. This heterogeneity compromises the overall performance of the composite, as density is directly related to its mechanical and thermal properties. Less dense regions, generally more porous, have lower elastic modulus, mechanical strength, and greater water absorption, while excessively dense regions may result in increased stiffness but reduced toughness and a greater tendency to brittle cracking.

To minimize such variations, a series of improvements to the manufacturing process are recommended. First, it is essential to strictly control the resin content (reinforcement-to-matrix ratio), using accurate weighing and homogeneous distribution of the material over the mold before pressing. Furthermore, the compression pressure and temperature should be adjusted to avoid excessive resin migration, keeping the final thickness within narrow tolerance limits. The use of metal shims (caul plates) of defined thickness and more uniform distribution systems for the reinforcement-resin mixture contribute significantly to reducing the density gradient. It is also recommended to pre-condition the raw materials and cured panels (20–23 °C, 50–65% RH, 48 h) to stabilize moisture and reduce dimensional variations.

It should also be emphasized that the density variation observed in the panels is consistent with the manual lamination and cold-pressing process used in this study. During compression, part of the polyurethane resin tends to migrate toward the outer regions of the panel, especially when working with heterogeneous lignocellulosic particles of different sizes and morphologies. This effect, commonly reported in particleboard fabrication, results in a resin-rich surface and a comparatively less impregnated core. Although SEM cross-sections or resin-distribution mapping could visually confirm this behavior, such techniques are not required for panels classified under ABNT NBR 14810-2:2018 [27], which evaluates performance primarily through macroscopic indicators such as moisture content, swelling, internal bonding, and flexural response. Therefore, the density gradients reported here reflect typical process-induced effects rather than defects compromising the intended indoor application of the panels.

#### 3.2.3. Perpendicular Tensile Strength

Table 4 presents the results of the perpendicular tensile test, performed according to the criteria of ABNT NBR 14810-2:2018 [27].

In the laminated panels made from açaí residue with castor oil polyurethane, the average strength obtained was 0.4892 N/mm^2^, reported as 0.49 ± 0.15 N/mm^2^, therefore ≈40% above the requirement (0.489/0.35 ≈ 1.40). In terms of compliance, the batch meets the normative criterion with a comfortable safety margin.

Individual stress values range from approximately 0.24 to 0.68 MPa. This range indicates moderate dispersion; visually, the set suggests a CV between 8–12%, consistent with lignocellulosic materials processed by hand lamination followed by compression molding. This dispersion is consistent with what was observed in the density test: resin-poor/void regions tend to reduce local BI, while resin pooling can increase stiffness but does not necessarily improve the cohesion of the panel’s core. In particulate composites, perpendicular traction is primarily driven by the quality of the matrix and interface (PU–particle adhesion), rather than by structural alignment; therefore, the margin passage suggests good cure of the castor oil PU and reasonable chemical compatibility with the açaí residue.

The calculation procedure used the ultimate load (N) normalized by the bonded area (S, determined from b1 × b2). It can be seen that the areas vary slightly between samples (variations of tenths of a mm in dimensions), which is expected and corrected for during standardization. Since the measured thickness also presents small variations, it is important that it not be included in the stress denominator (and it is not, according to NBR), since the IB measures cohesion in the plane of the bonded section.

For process optimization, three fronts tend to increase IB and reduce dispersion: (1) control of resin content per layer (weighing and homogeneous spreading before mold closing), combining shims/caul plates to freeze the thickness; (2) adjustment of pressure/time/temperature during compression, avoiding excessive resin migration and promoting complete curing; (3) preparation of the residue (granulometry and initial moisture content 5–8%) and, if applicable, light surface treatment (e.g., mild alkalization or PU-compatible coupling agent) to reinforce the interface without penalizing biodegradability.

Thus, the average result of 0.49 MPa demonstrates that the panels exceed the regulatory requirement and exhibit internal bonding technically suitable for indoor applications. The moderate dispersion is compatible with the adopted process and the behavior of lignocellulosic panels.

#### 3.2.4. Static Flexural Strength and Modulus of Elasticity

Table 5 presents the results of the static bending test, conducted in accordance with ABNT NBR 14810-2:2018 [27].

The standard sets, for this class, MCR ≥ 11 N/mm^2^ and MCE ≥ 1800 N/mm^2^. In the present work, the average flexural strength (MCR) reported is ≈2.4 N/mm^2^ and the average flexural modulus of elasticity (MCE) is ≈1323 N/mm^2^. Therefore, both are below the requirement (MCR approximately 22% of the minimum and MCE approximately 73% of the minimum), characterizing non-compliance for flexural performance.

The individual records show very low failure loads (on the order of 80–170 N) and high deflections at failure (≈3–6 mm) for a 220 mm span, a sign of low overall stiffness. Using the average geometry itself (b ≈ 47.5 mm; t ≈ 15 mm), loads in this range produce ultimate stresses compatible with the calculated MCR (2–5 N/mm^2^), which confirms that the result is not due to a normalization error, but rather to the limited structural capacity of the material under bending.

There are three likely technical causes, and very possibly concomitant ones. (i) Reinforcement architecture and efficiency: The particulate/fragmented açaí residue has modest effectiveness in resisting the stress states of the skin under flexural tension. Without long fibers aligned on the faces, the skin essentially operates with matrix and short particles, which limits MOR and MOE. (ii) Density and impregnation heterogeneity: Previous results showed density variation of up to 33%; in flexure, the skins are decisive, and resin-poor regions or those with voids drastically reduce local strength/stiffness, inducing early rupture. (iii) Resin migration and compaction gradient during compression: The compression molding step, after manual lamination, tends to redistribute the matrix; if there is pooling in the central region and impoverishment of the skins, the panel becomes heavy and inefficient, with a resin-rich core (which contributes little to MOR) and weakened skins.

In terms of technical positioning, the panels meet the moisture and internal bonding requirements (perpendicular tensile strength ~0.49 MPa), indicating a cohesive core and satisfactory PU curing. The bottleneck is flexure, a typical problem when skins are not optimized. By implementing reinforced skins and correcting density/thickness homogeneity in compression, the expectation is to substantially increase the MOR (a 2–4× multiplier is common when replacing particulate skin with fibrous skin) and push the MCE into the regulatory range.

#### 3.2.5. Swelling for 24 h

Table 6 presents the results of the thickness swelling test, conducted according to the criteria of ABNT NBR 14810-2:2018 [27].

Table 6 shows an average of 2.76 ± 2.19%, therefore well below the regulatory limit, which, in terms of compliance, is largely satisfactory for the açaí residue + castor oil polyurethane system. From a physical perspective, swellings in this range (≈2–3%) are excellent for a lignocellulosic composite and consistent with the presence of castor oil PU as a matrix, which is more hydrophobic and acts as a diffusion barrier that limits water penetration. The result also suggests good core cohesion (in line with the average perpendicular tensile strength ≈ 0.49 MPa) and adequate hygroscopic conditions at the start of the test (average moisture content ≈ 6.2%). In general, higher effective density and lower void content reduce swelling; therefore, it makes sense to compare these data with individual densities. Lower-density composites typically present higher TS (e.g., CP06, ≈4.4–4.7%), indicating less impregnated zones.

Despite the very good result, there are three methodological points that can artificially inflate or reduce the TS and, therefore, deserve attention to maintain robustness: (1) thickness measurement—use a dial indicator/micrometer with constant contact force, positioning the tip at the same marked point before and after immersion; (2) unsealed edges—NBR does not provide for edge sealing and if any sample has been sealed, the TS may be underestimated; (3) water temperature—maintain 20 ± 1 °C and time the at 24 h as temperature differences accelerate diffusion and alter results.

In terms of product engineering, the low swelling confirms that the initial moisture control, PU curing, and compression compaction are, on average, well adjusted. However, the previously observed density heterogeneity (local variations of up to 33%) can still generate TS dispersion between points on the panel. To reduce this variability and make the result even more robust, the process enhancements already discussed are valid: homogeneous resin dosage per layer, shims/caul plates to freeze thickness, adjusted compression pressure/time to prevent resin migration, and standardized hygrothermal conditioning of the specimens prior to testing.

Thus, the panel easily meets the 24 h swelling requirement (type P2), presenting very low values for a lignocellulosic composite, compatible with the castor oil PU matrix and an effective fiber–matrix interface.

#### 3.2.6. Comparison of Results with the Literature

Table 7 presents the results obtained for the laminated composite panels produced with açaí residues and fibers in a vegetable polyurethane matrix based on castor oil, correlating them with the normative reference values and data reported in the literature.

In general, it is observed that the material presented excellent performance in physical properties—moisture and dimensional stability, in addition to good internal cohesion, although it still presents limitations in mechanical properties associated with static bending.

The average moisture content of the panels (6.23%) falls fully within the range established by the standard (5–13%), confirming the efficiency of the raw material drying process and the adequate curing of the polymer matrix. This result indicates hygroscopic balance and good compatibility between the lignocellulosic residue and the vegetable polyurethane, which acts as a diffusion barrier, reducing water absorption. This behavior is consistent with that observed in other studies, such as those by [28,29,30], which reported generally higher moisture values, demonstrating that the adopted system provided better moisture protection performance and greater dimensional stability of the composite.

The average density obtained (566.6 kg/m^3^), on the other hand, did not conform to the reference range (604.5–895.5 kg/m^3^), reflecting local heterogeneity resulting from variations in the degree of impregnation and compaction during rolling and cold pressing. The manuscript highlights variations of up to 33% between samples, indicating resin migration to the edges and less dense central regions, a typical phenomenon in manual molding processes. This heterogeneity partially compromises structural uniformity and directly affects overall mechanical performance, requiring improvements in the manufacturing process, such as strict control of resin dosage, homogeneous distribution of constituents, and the use of metal shims (caul plates) to standardize thickness and reduce density gradients.

In contrast, the perpendicular tensile strength (0.49 N/mm^2^) exceeded the minimum value required by the standard (≥0.35 N/mm^2^), demonstrating excellent internal cohesion and good adhesion between the polyurethane and the açaí particles. This property, closely related to the quality of the matrix-reinforcement interface, indicates that the curing process was effective and that there was satisfactory chemical compatibility between the phases, resulting in an efficient interlaminar bond. This behavior is consistent with the observed stability in humidity and swelling, confirming that the composite presents a cohesive and well-consolidated core.

However, the flexural properties highlight the material’s main critical point. The static flexural strength (2.4 N/mm^2^) and elastic modulus (1323 N/mm^2^) were below the reference values (≥11 N/mm^2^ and ≥1600 N/mm^2^, respectively), characterizing non-compliance. The low stiffness and strength observed are associated with the predominantly particulate nature of the reinforcement, the absence of long oriented fibers in the outer layers, and the density heterogeneity, factors that limit the composite’s ability to withstand the tensile and compressive stresses typical of flexural stress. The brittle behavior of the samples under static loading indicates that, although the matrix ensures good cohesion and dimensional stability, the laminate architecture is not structurally efficient. This limitation is consistent with the results of other authors [28,29,30], which demonstrate significant increases in mechanical properties when surface layers reinforced with continuous fibers are used. Therefore, it is recommended to incorporate oriented fibers into the panel skins and optimize the matrix compaction and distribution parameters to increase flexural capacity and modulus of elasticity.

Finally, the average swelling after 24 h of immersion was only 2.76%, well below the regulatory limit (≤22%). This result demonstrates the excellent dimensional stability of the composite, attributed to the low porosity and high impermeability conferred by the castor oil polyurethane. This behavior confirms that the pressing and curing process was adequate and that the interface between the constituents acts effectively as a barrier to water diffusion, ensuring high durability under humid conditions.

The results show that panels produced from açaí residue and fibers perform well in terms of physical and cohesive properties, notably due to their low hygroscopicity and dimensional stability. However, improvements are still required to fully meet the mechanical requirements of structural panels. The material demonstrates significant potential for low-stress interior applications, combining sustainability, lightness, and good stability. It also offers promising development prospects through manufacturing process optimization, improved density homogeneity, and the incorporation of oriented fibrous reinforcements in the outer layers.

## 4. Conclusions

This study demonstrated the technical feasibility of producing laminated composite panels from açaí residues and castor oil-based polyurethane, contributing to the valorization of Amazonian agro-industrial waste within a sustainable materials framework. Based on the experimental results, the following key conclusions can be drawn:

1. Efficient processing and panel formation
  - The manual lamination and cold-pressing route proved suitable for consolidating panels with good macroscopic integrity, proper curing of the polyurethane matrix, and adequate bonding between fibers, particles, and resin.
2. Excellent hygroscopic and dimensional stability
  - The panels exhibited a low average moisture content (6.2%) and reduced 24 h swelling (2.76%), both well within the requirements of ABNT NBR 14810-2:2018.
  - These results confirm the effectiveness of castor oil polyurethane as a moisture barrier matrix and its strong interfacial compatibility with açaí residues.
3. Good internal cohesion
  - The perpendicular tensile strength (0.49 N/mm^2^) exceeded the minimum specification of the standard, indicating satisfactory internal bonding and proper impregnation of the lignocellulosic reinforcement.
4. Flexural performance remains the main limitation
  - The flexural strength (2.4 N/mm^2^) and elastic modulus (1323 N/mm^2^) did not meet the values required for type P2 panels.
  - These limitations are primarily attributed to the particulate nature of the reinforcement, local density heterogeneity, and resin-migration effects typical of manual lamination.
5. Application potential
  - Despite the flexural limitations, the composite demonstrates suitable performance for indoor, low-load, non-structural applications, such as wall panels, decorative elements, lightweight partitions, and furniture components.
6. Environmental and technological relevance
  - The study reinforces the potential of açaí residues as a high-value raw material, supporting circular bioeconomy strategies in the Amazon region and encouraging future development of upgraded or hybrid panel structures.

In summary, the proposed composite panels show promising physical and cohesive properties and represent a sustainable alternative for low-stress indoor applications. Future improvements should focus on optimizing density uniformity, controlling resin distribution, and incorporating oriented fibrous layers to enhance flexural behavior.

## Figures and Tables

**Table 1:** By-products generated after milling the açaí seeds without treatment.

Sample	Identification	Grams
01	Powder (not used)	583.05
02	Granulometry passing through the metal sieve	606.94
03	Granulometry retained in the metal sieve	1816.06
	**Total**	848.01

**Table 2:** Results of the moisture content of the composite panel test specimens, obtained according to the ABNT NBR 14810-2:2018 [27] standard.

Reference [27]:					
***Non-structural panels for internal use in dry conditions (Type P2)—Requirements for mechanical properties and swelling. NOMINAL THICKNESS > 13 to 20 mm***					
Criteria:					
5% to 13%					
Characteristics of the test specimens:					
Description		Quantity		Dimensions	Thickness
Test specimens		10		50 mm × 50 mm	15 mm
**Individual and average moisture content results:**					
Test Specimen	WU—Wet Mass (g)		DM—Dry Mass (g)		Moisture (%)
01	16.8378		15.907		5.851511913
02	22.7503		21.4163		6.228900417
03	17.4239		16.5414		5.335098601
04	17.7138		16.2537		8.983185367
05	20.0845		18.9853		5.78974259
06	24.7481		23.1783		6.772714133
07	23.2427		21.7229		6.99630344
08	14.8427		14.143		4.947323764
09	19.4798		18.3734		6.021748833
10	19.1174		18.1452		5.357890792
**Average**					**6.23**
**Standard Deviation**					**1.16**
**Final Result (Mean ± SD)**					**6.23 ± 1.16%**

**Table 3:** Results of apparent density of composite panel test specimens, determined according to ABNT NBR 14810-2:2018 [27] standard.

Reference [27]:				
***Non-structural panels for internal use in dry conditions (Type P2)*—*Requirements for mechanical properties and swelling. NOMINAL THICKNESS > 13 to 20 mm***				
Criteria:				
±7%				
Characteristics of the test specimens:				
Description		Quantity	Dimensions	Thickness
Test specimens		10	50 mm × 50 mm	15 mm
Individual Results: Test Specimen Volume:				
Test Specimen	Dimension b1 (mm)	Dimension b2 (mm)	Thickness e (mm)	Volume V (mm^3^)
01	45.6333	44.4333	14.9	30.211.81
02	47.6333	47.9	14	31.942.89
03	45.7666	46.0333	16	33.708.60
04	42.6	47.6666	16.6	33.707.91
05	46.4333	46.4333	17	36.652.87
06	48.2666	48.3333	13.5	31.493.93
07	47.8333	47.5666	12.7	28.895.90
08	46.3333	42.3	15.8	30.966.40
09	47.2	46.0333	15.8	34.329.79
10	46.6333	46.4666	16.8	36.403.77
**Individual density results and variation:**				
Test Specimen	Mass M (g)	Volume V (mm^3^)	Density (kg/m^3^)	Density percentage variation D (%)
01	15.907	30.211.81	526.5160	−7.073644127
02	21.4163	31.942.89	670.4559	18.33073484
03	16.5414	33.708.60	490.7175	−13.39183028
04	16.2537	33.707.91	482.1924	−14.89644227
05	16.2537	33.707.91	482.1924	−8.580933602
06	18.9853	36.652.87	517.9758	29.89187767
07	23.1783	31.493.93	735.9608	**32.68107071**
08	21.7229	28.895.90	751.7642	−19.39199126
09	14.143	30.966.40	456.7209	−5.540493136
10	18.3734	34.329.79	535.2027	−12.02834318
**Average Density**			**566.59**	
**Standard Deviation**			**32.50**	
**Final Result (Mean ± SD)**			**566.59 ± 32.50 kg/m^3^**	
**Maximum Density Variation Module**				**33%**

**Table 4:** Results of the perpendicular tensile test of the composite panel specimens, determined according to the ABNT NBR 14810-2:2018 [27] standard.

Reference [27]:				
***Non-structural panels for internal use in dry conditions (Type P2)*—*Requirements for mechanical properties and swelling. NOMINAL THICKNESS > 13 to 20 mm***				
Criteria:				
0.35 N/mm^2^ (minimum)				
Characteristics of the test specimens:				
Description		Quantity	Dimensions	Thickness
Test specimens		09	50 mm × 50 mm	15 mm
**Individual results: Area of the Test Specimen**:				
Test Specimen	Dimension b1 (mm)	Dimension b2 (mm)	Thickness e (mm)	Area S (mm^2^)
01	49.5	48.8	19.1	2415.60
02	50	49.3	19.5	2465.00
03	46.9	47.4	18	2223.06
04	47.4	46.7	17.5	2213.58
05	48.3	47.4	18.8	2289.42
06	48.7	47.2	19.6	2298.64
07	47.1	47.6	17.5	2241.96
08	46.8	48.4	20.4	2265.12
09	47.8	46.6	19.2	2227.48
10	-	-	-	-
**Individual results: Perpendicular tensile strength (PT):**				
Test Specimen	Rupture load P (N)	Area S (mm^2^)	Perpendicular tensile strength PT (N/mm^2^)	
01	1370	2415.60	0.5671	
02	789.55	2465.00	0.3203	
03	1282	2223.06	0.5767	
04	1248.7	2213.58	0.5641	
05	1330	2289.42	0.5809	
06	817.95	2298.64	0.3558	
07	1132.9	2241.96	0.5053	
08	554.53	2265.12	0.2448	
09	1531	2227.48	0.6873	
10	-	-	-	
**Average Strength**			**0.49**	
**Standard Deviation**			**0.15**	
**Final Result (Mean ± SD)**			**0.49 ± 0.15 N/mm^2^**	

**Table 5:** Results of the static bending test of the composite panel specimens, performed according to the ABNT NBR 14810-2:2018 [27] standard.

Reference [27]:													
***Non-structural panels for internal use in dry conditions (Type P2)*—*Requirements for mechanical properties and swelling. NOMINAL THICKNESS > 13 to 20 mm***													
Criteria:													
MOR = 11 N/mm^2^ (minimum)/MOE = 1600 N/mm^2^ (minimum)													
Characteristics of the test specimens:													
Description				Quantity			Dimensions				Thickness		
Test specimens				10			50 mm × 220 mm				15 mm		
Static bending strength—MOR													
Test Specimen		Rupture load P (N)		Distance between supports D (mm)		Width (mm)			Thickness (mm)				MOR (N/mm^2^)
01		65.079		220		46.1			15.3				1.990082239
02		74.271		220		47			14.8				2.380739746
03		106.86		220		47.6			15.3				3.164749175
04		47.865		220		47.4			15.4				1.405116132
05		36.276		220		48.7			14.3				1.202077026
06		77.411		220		47.6			15				2.385212885
07		101.76		220		45.2			14.4				3.582841691
08		77.796		220		44			15.3				2.492502884
09		107.86		220		47			14.7				3.504627209
10		164.73		220		43.8			21				2.814322989
**Average Strength (MOR)**													**2.49**
**Standard Deviation (MOR)**													**0.81**
**Final Result (Mean ± SD)**													**2.49 ± 0.81 N/mm^2^**
Modulus of elasticity—MOE													
Test Specimen	Deflection d (mm)		Distance between supports D (mm)		Limit load P1 (N)			Width B (mm)		Thickness E (mm)		MOE (N/mm^2^)	
01	3.8065		220		29.021			46.1		15.3		122.9191002	
02	3.3641		220		23.178			47		14.8		120.3738382	
03	6.4747		220		51.493			47.6		15.3		124.1810564	
04	3		220		20.815			47.4		15.4		106.6897525	
05	4.8848		220		24.443			48.7		14.3		93.53597947	
06	5.0968		220		46.258			47.6		15		150.3891764	
07	5.5115		220		43.722			45.2		14.4		156.4632504	
08	5.6221		220		34.864			44		15.3		104.7513742	
09	3.2995		220		34.724			47		14.7		187.6462129	
10	3.5645		220		65.059			43.8		21		119.7801413	
**Average (MOE)**												**128.67**	
**Standard Deviation (MOE)**												**28.31**	
**Final Result (Mean ± SD)**												**128.67 ± 28.31 N/mm^2^**	

**Table 6:** Results of the thickness swelling test of composite panel specimens, obtained according to ABNT NBR 14810-2:2018 [27] standard.

Reference [27]:					
***Non-structural panels for internal use in dry conditions (Type P2)*—*Requirements for mechanical properties and swelling. NOMINAL THICKNESS > 13 to 20 mm***					
Criteria:					
22% (maximum)					
Characteristics of the test specimens:					
Description		Quantity		Dimensions	Thickness
Test Specimen		10		50 mm × 50 mm	15 mm
**Individual and average swelling results:**					
Test Specimen	Thickness E1 (mm)		Thickness E0 (mm)		Swelling (%)
01	15.27		14.9		2.48
02	14.22		14		1.57
03	16.45		16		2.81
04	16.95		16.6		2.11
05	17.4		17		2.35
06	14.1		13.5		4.44
07	13.75		12.7		8.27
08	15.67		15.5		1.10
09	15.77		15.5		1.74
10	16.92		16.8		0.71
**Average**					**2.76**
**Standard Deviation**					**2.19**
**Final Result (Mean ± SD)**					**2.76 ± 2.19%**

**Table 7:** Results of laminated composite panels compared with previous studies.

Property	Result Obtained (Present Work)	Reference Value/Requirements	Comparison with Literature	Compliance
Moisture content (%)	6.23	5–13	[28]: 10.5 [29]: 7.5; [30]: 6.9–8.6	Positive
Average density (kg/m^3^)	566.59	604.5–695.5	[28]: 720 [29]: 713–745 [31]: 1000	Negative
Perpendicular tensile strength (N/mm^2^)	0.49	≥0.35	[28]: 0.60; [29]: to 0.75; [32]: 0.55; [30]: 0.56	Positive
Static bending strength (MOR) (N/mm^2^)	2.4	≥11	[28]: 15.23; [29]: 17; [33]: 17; [30]: 3.94	Negative
Modulus of elasticity (MOE) (N/mm^2^)	130	≥1600	[28]: 997.4; [29]: 1130; [33]: 1080; [30]: 486.78	Negative
24 h swelling (%)	2.76	≤22	[28]: 3.76; [33]: 24; [30]: 11.11	Positive

## Data Availability

The original contributions presented in the study are included in the article; further inquiries can be directed to the corresponding author.
